# Study on the effects of environmental factors on enzyme activities during growth of *Hypsizygus marmoreus*

**DOI:** 10.1371/journal.pone.0268107

**Published:** 2022-08-31

**Authors:** Hongpeng Yang, Meige Lei, Liang Huang, Yu Wang, Ning Sun, Litong Ban, Xufeng Wang, Hongyang Zhang

**Affiliations:** 1 College of Agronomy & Resources and Environment, Tianjin Agricultural University, Tianjin, China; 2 Collage of Agriculture, Nanjing Agricultural University, Nianjin, China; 3 Tianjin Hong Bin He Sheng Agricultural Technology Development Co. Ltd., Tianjin, China; 4 Tianjin Bin Hai De Sheng Agricultural Technology Co. Ltd., Tianjin, China; ICAR-Directorate of Mushroom Research, INDIA

## Abstract

The sensitivity of *Hypsizygus marmoreus* to environmental factors such as temperature, humidity, illumination and CO_2_ concentration varies greatly in different growth stages. In this paper, the effects of various environmental factors on the growth and development of *H*. *marmoreus* were investigated by measuring the enzyme activities of *H*. *marmoreus* at different growth stages under different microenvironment conditions in the mushroom room, so as to confirm the influence mechanism of environmental factors on the growth of *H*. *marmoreus*. The results showed that at budding stage xylanase and laccase were found significantly positively correlated with CO_2_ concentration and light intensity, and dramatically negatively correlated with humidity while carboxymethyl cellulose and manganese peroxidase were markedly positively correlated with humidity, and significantly negatively correlated with CO_2_ concentration and light intensity. On the other hand, in mature fruit bodies xylanase activity was found significantly positively correlated with CO_2_ concentration and light intensity, and dramatically negatively correlated with humidity while manganese peroxidase activities were found significantly positively correlated with humidity, and dramatically negatively correlated with light intensity. The activity of β-glucosidase in budding and mature fruiting bodies was markedly negatively correlated with CO_2_ concentration and significantly positively correlated with humidity.

## Introduction

*Hypsizygus marmoreus* (Peck) H.E. Bigelow belongs to Basidiomycotina, Hymenomycetes, Agaricales, Tricholomatacese, *Hypsizigus* [1]. Since commercial cultivation of *H*. *marmoreus* began in Japan in the 1970s, it has become one of the most important edible fungi varieties in East Asia [2]. Humidity, CO_2_ concentration and light time requirements varies with different growth stages in *H*. *marmoreus*. Its cultivation environment is a complex non-linear and time-varying system. The effects of various environmental factors on the qualities and yield of fruiting bodies of *H*. *marmoreus* cannot be neglected. If environmental factors are not effectively controlled and maintained in the stage of primordium formation and fruiting body growth, mushroom production may be seriously affected, and can be the bottleneck of economic growth of enterprises. Previous studies found that the growth condition of *H*. *marmoreus* on the 6th shelf of the same mushroom house is the best with a biological conversion rate of 65.96%. Controlling environmental factors such as temperature 15.3°C, relative humidity of 89.5%, light intensity of 870 LX and CO_2_ concentration of 2029 mg·kg ^-1^ could significantly improve the biological conversion rate of *H*. *marmoreus* [3]. This indicates significant effect of environmental factors on the growth of *H*. *marmoreus*.

The survival and reproduction of mushrooms is related to a number of factors, which may act individually or have interactive effects among them. C/N ratio of culture medium, minerals, pH value, water content, and other conditions are considered to be chemical, physical and biological factors that are linked to mushroom production [4]. Earlier studies on the influence of environmental factors on the cultivation of mushroom showed that temperature, humidity, CO_2_ concentration and wind speed directly affected the yield and quality of mushroom [3, 4]. According to Chang and Miles, the appropriate relative humidity during spawn-running and mycelia stimulation should encompass a range between 60–75% and 85–97%, respectively, enabling a satisfactory growth of *Pleurotus spp*. [4, 5]. During spawn-running, it is important to keep CO_2_ concentration at 2000–2500 mg L^-1^. After the completion of spawn-running and mycelial stimulation, fruit bodies were allowed to develop at CO_2_ concentration 1500–2000 mgL^-1^ [4, 6]. High concentrations of carbon dioxide in the air can produce mushrooms with thick and short stipes. Therefore, during the fruiting stage, a reduction in CO_2_ concentration is required with an increase in O_2_ [4]. In general, the photoperiod of mycelial stimulation to promote mushroom fruit bodies formation should be sufficient to read a sheet of paper (200–640 lux 8–12 h a day^-1^) at a temperature compatible with the mushroom [7]. In the complete absence of light, oyster mushrooms will form no cap but stipes (mushroom stalks) will be a coral-like structure. Environments that have a lot of light may cause paleness, deformations, elongated stipe and reduction of pileus coloration. Photoperiod is not necessary to induce the primordium formation but it is needed for fruiting body production. Studies have shown that the vitamin D_2_ content in mushrooms (*Agaricus bisporus and Pleurotus ferulae*) can be significantly increased in a short period of time by using a wide spectrum (100–800 nm) lamp in the form of high intensity pulses [4]. Recent studies reported blue-light treatment significantly up-regulated gene expression involved in glycolysis/gluconeogenesis, the pentose phosphate pathway and the peroxisome in the pileus [8]. These suggest that light may affect the enzymes activities involved in metabolic regulation during mushroom growth and development. During the growth and development of mushroom, the absorption and utilization of nutrients cannot be separated from the corresponding enzymes, such as xylanase (EC 3.2.1.8), carboxymethyl cellase (CMCase, EC 3.2.1.4), laccase (EC 1.10.3.2), manganese peroxidase (MnP, EC 1.11.1.13), β-glucosidase (EC 3.2.1.21), and so on. The study of correlation between these enzymes and the environmental conditions is very important.

In order to provide a basis for the accurate control of *H*. *marmoreus* in industrial cultivation, the effects of environmental factors such as humidity, CO_2_ and light intensity on degrading enzymes in budding and ripening stages of *H*. *marmoreus* were studied in this paper, so as to confirm the influence mechanism of environmental factors on the growth of *H*. *marmoreus*. The results of this study will play a guiding role in the large-scale cultivation of *H*. *marmoreus* with high efficiency and economy.

## Materials and methods

### Details and treatments

#### Reagents

2, 2ʹ-azinobis-(3-ethyl-benzothiazoline-6-sulfonicacid) diammonium salt (ABTS), salicylic acid glycoside, birchwood xylan were purchased from Sigma Chemical Company. All other chemical reagents were analytically pure and were purchased from Tianjin Fang Chuan chemical reagent Technology Co., Ltd.

#### Materials

**Fig 1:**
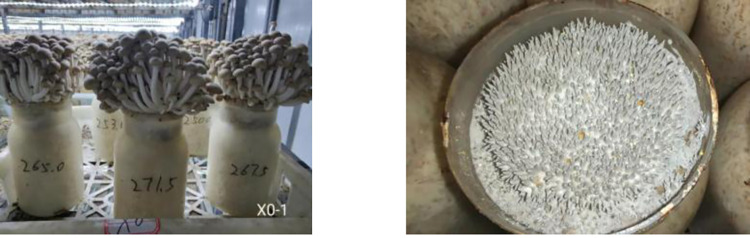
The fruiting body samples at bud stage and mature stage.

The cultivation materials of *H*. *marmoreus* consist of cottonseed hull, corn flour, corncob, wheat bran, rice bran and sawdust. The fruiting body samples at bud stage and mature stage shown in Fig 1 were cultivated in the mushroom house of Tianjin Hong bin He sheng Agricultural Technology Development Co., Ltd.

#### Sampling method

**Fig 2:**
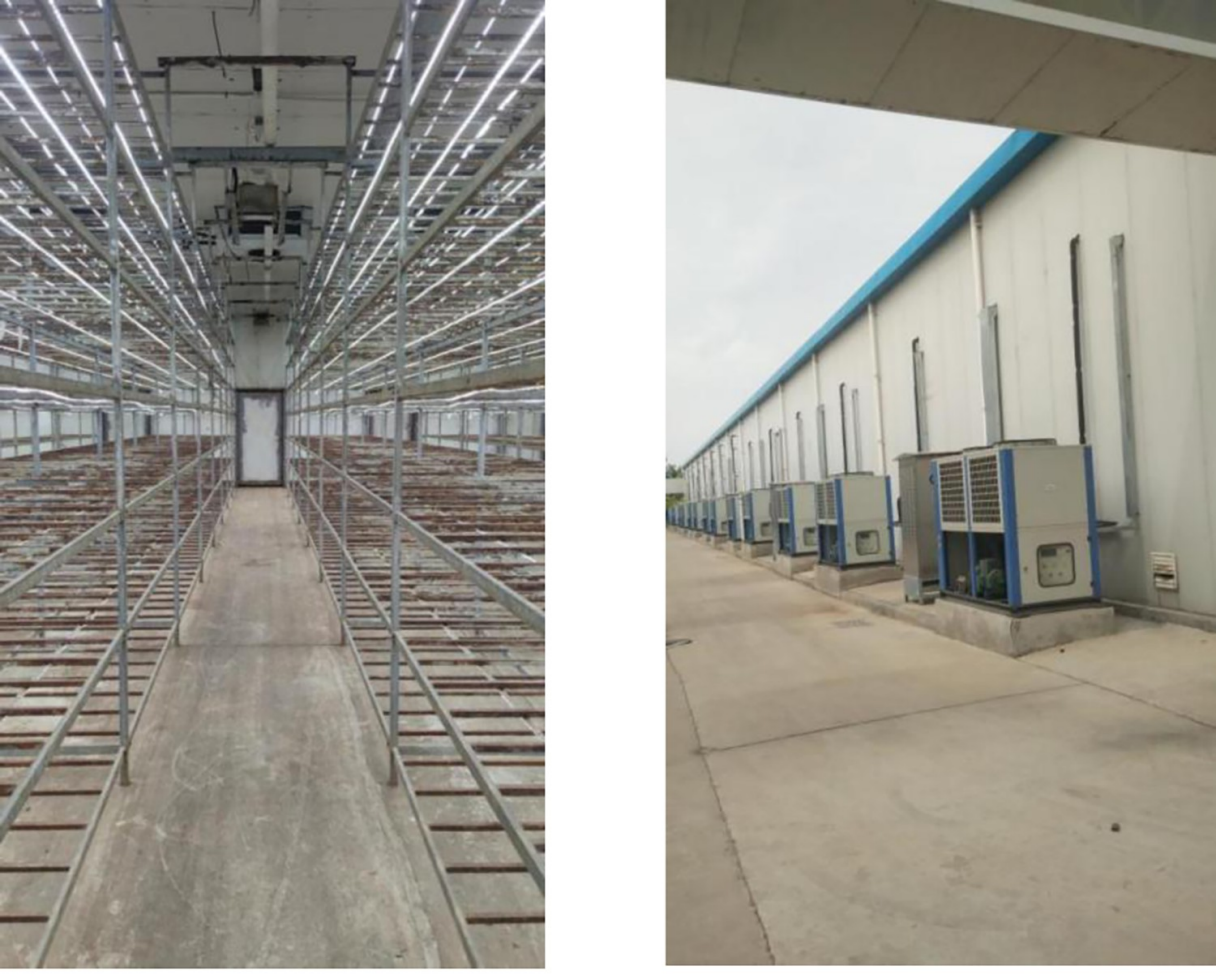
Photo of mushroom house.

**Fig 3:**
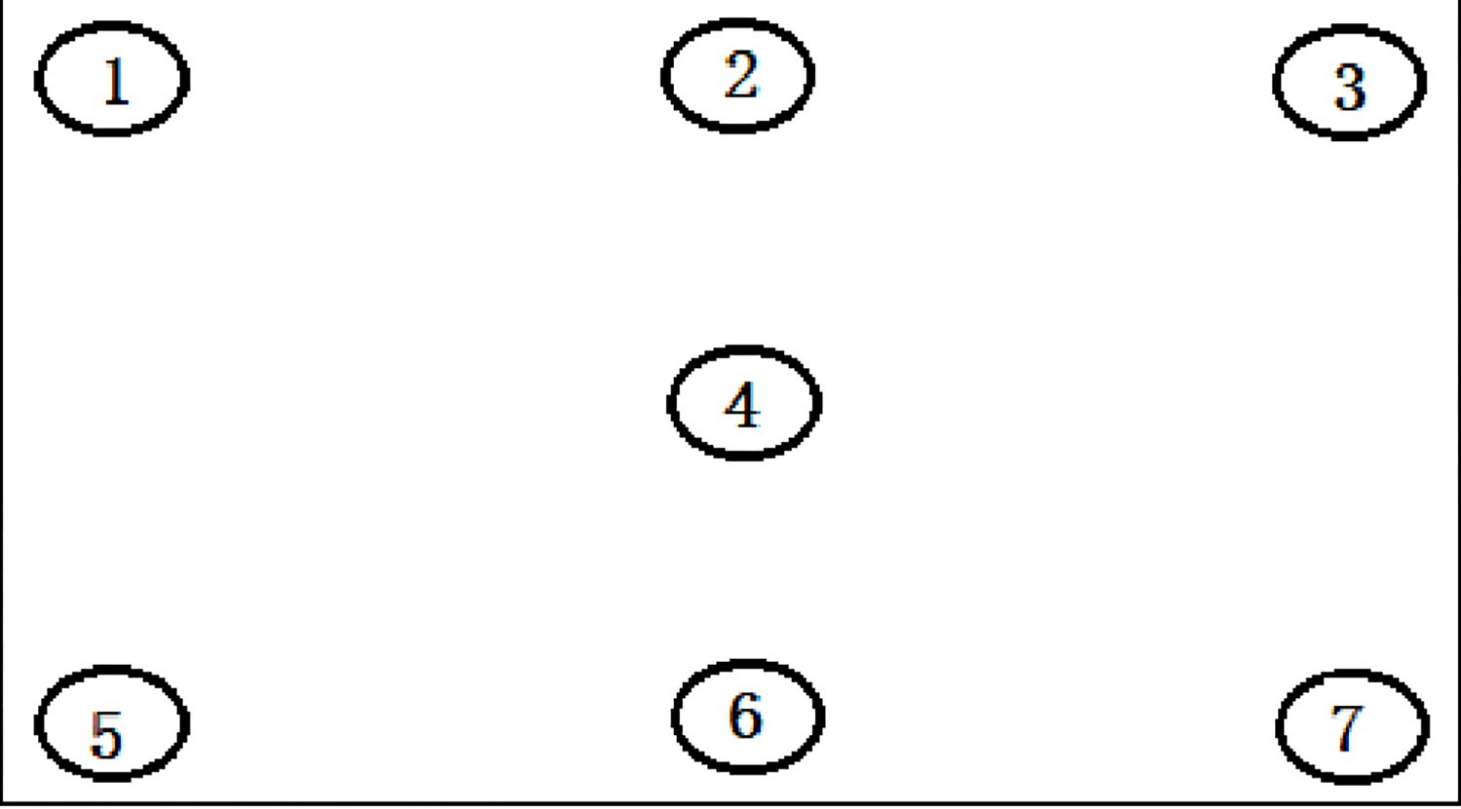
Sampling plan for mushroom house.

Bottle cultivation method was used with a batch size of 60000 bottles. Each bottle of 1100 ml volume consisted of 230±2g and 660±5g dry and wet raw material, respectively. Stepped high pressure sterilization method was used to sterilize, and kept at 121°C for 90 min. The cultivation period was 121–123 days. A mushroom house was randomly selected to measure environmental parameters and collect samples (Fig 2). There are six tiers in the mushroom house of size (18mx13mx4.8m) at a gap to 55 cm between tiers. Samples of cultivation bottles were collected from 1^st^, 3^rd^ and 6^th^ tier. Three positions were selected in each tier (Left, right and middle positions) (Fig 3). The first tier is the bottom floor and the sixth tier is the highest tier. Samples were collected from each sampling point and mixed for enzyme activity determination.

#### Measurement of humidity, CO_2_ and light intensity

Humidity was measured by hygrometer at different places of different tiers (Youlide UNI-T), CO_2_ was measured by BW GasAlert Micro 5IR, and light intensity was measured by portable light intensity tester (Asensetek ALP-01). The air supply system is designed to supply fresh air through the roof of the mushroom house and extract air from the ground layer (open and close at regular intervals). After the mycelium recovery period, light stimulation was given for 20 days, and the light duration and frequency were gradually extended according to the procedure (Table 1). Only the light intensity was measured in this study, not the illumination time and time interval. The CO_2_ concentration in the mushroom house was regulated by a ventilator 35 cm above the ground. The humidity control in different growth stage is controlled by the humidifier, which is turned on at a certain interval and turned off after humidification for a certain period of time. Humidity controlled at 90%±5%.

**Table 1:** Control parameters of mushroom house.

Variety	Stage	Temperature	Humidity management (On /off)	Lighting (on/off)	Ventilator (open /close)
*H*. *marmoreus*	1~4 d	14.2~15.6°C	10~8 min/5 min	No	5 min/10 min
	5~8 d	14.1~15.7°C			10 min/60~70 min
	9~14 d	14.5~15.3°C		10 min/90 min	10/50 min~60 min
	15~17 d	14.5~15.2°C	10~5 min/18~28 min	20 min/60 min	10 min/50 min
	18~20 d	14.6~15.1°C		30 min/40 min	10 min/50 min
	21d	fruiting	3 min/20 min	No	5 min/60 min

#### Enzyme activity determination

*Preparation of crude enzyme solutions*. Samples collected were weighed 10 g and placed in a mortar. After adding 30 mL ice-water mixture and 3 g quartz sand, the samples were ground into homogenate and transferred into 10 mL centrifugal tube. The samples were centrifuged for 10 min in a cryogenic centrifuge at 5366.4×g, and the supernatant was refrigerated at 4°C.

*Determination of laccase activity*. The changes of absorbance in 3 minutes at 420 nm were determined by adding 50μL crude enzyme solution, 150μL acetic acid-sodium acetate buffer (pH 5.0, 50mmol·L^-1^), 100μL 1mmol·L^-1^ ABTS into 96-well plate. Three replicates were measured for each sample. The enzyme activity was defined as the amount of enzyme required to oxidize 1 micromol ABTS/min. Activity of laccase was calculated by pressing down formula [9–11].


$$U/L=\frac{\Delta OD\times V_{1}}{\varepsilon_{420}\times V_{2}\times \Delta t}\times 10^{6}$$

ΔOD is the difference of absorbance between the start and end. V_1_ is the total volume of enzymatic reaction mixture. V_2_ is the amount of enzymatic solution added. Molar extinction coefficient (*ε*) of ABTS is 36000 M^-1^·cm^-1^ (420nm in acetic acid-sodium acetate buffer). Δt is the reaction time (3 min).

*Determination of carboxymethyl cellulose activity*. Citric acid buffer (pH 5.0, final concentration 50mmol·L^-1^) containing 1.5 mL of 1% CMC-Na as reaction substrate and 0.5 mL crude enzyme solution was placed in 25 mL plugged test tube. 3 mL DNS (3, 5-dinitrosalicylic acid) was added immediately after 30 minutes incubation at 50°C in water bath, boiled for 10 minutes, and cooled under running water to room temperature. The volume is fixed to 25 mL by distilled water. After shaking, 200 μL was taken out in 96-well plate. The absorbance at 540 nm was measured by microplate reader (PerkinElmer Enspire). The crude enzyme solution boiled for 10 minutes was used as the control group. Enzyme activity was defined as the amount of enzyme required to catalyze the substrate to produce 1 μmol glucose per minute as a unit of enzyme activity [10, 12, 13]. The difference between the measured absorbance and the control was substituted into the following formula to obtain the enzyme activity.


$$U/L=\frac{\frac{\Delta OD-n}{m}\times V_{0}\times \frac{V_{2}}{V_{2}}}{M_{\mathrm{glucose}}\times \Delta t\times V_{2}}$$

Among them, ΔOD value is the difference between the absorbance value measured in treatment and that measured in control group, which is substituted into the standard curve equation to obtain the concentration of glucose. V_0_ is the volume after final dilution (25mL). V_1_ is the total volume of the enzyme reaction mixture (the total volume of the enzyme solution and the reaction substrate). M_glucose_ is the molar mass of glucose used in the experiment, Δt is the time of enzymatic reaction (30min), n is the intercept, m is the slope and V_2_ is the volume of enzymatic liquids added. V_1_ divided by V_2_ is the dilution multiple.

*Determination of xylanase activity*. A volume of 0.1 mL crude enzyme solution was added into 25 mL plugged test tube with 0.9 mL of 1% birchwood xylan (0.05mol·L^-1^ citric acid buffer, pH 5.0) as reaction substrate. The reaction system was incubated at 50°C for 30 minutes in a water bath, then removed and immediately added 3 mL DNS reagent, and boiled for 10 minutes. After cooling to room temperature under water, the volume is fixed to 25 mL by distilled water. The absorbance value at 540 nm was determined by microplate reader (PerkinElmer Enspire), using 200 μL in a 96-well enzyme-labeling plate. The inactivated crude enzyme solution boiled for 10 minutes beforehand was used as control group. The unit of enzymatic activity is defined as the amount of enzymes required to decompose 1 μmol xylan per minute [13, 14].


$$U/L=\frac{\frac{\Delta OD-n}{m}\times V_{0}\times \frac{V_{1}}{V_{2}}}{M_{xylose}\times \Delta t\times V_{2}}$$

M_xylose_ is the relative molecular weight of xylose used in the formula, and the other items are the same as the formula for calculating the activity of CMCase.

*Determination of manganese peroxidase activity*. A volume of 2 mL of 50 mmol·L^-1^ lactic acid-sodium lactate buffer solution (pH 4.5), 0.1 mL of 1.6 mmol·L^-1^ MnSO_4_ solution and 0.8 mL crude enzyme solution were added to 10 mL centrifugal tube. The reaction was initiated by adding 0.1 ml 1.6 mmol·L^-1^ H_2_O_2_ at room temperature. After 3 minutes, the tube solution was quickly put into ice water. 200 μL reaction solutions were added into 96-well plate and the absorbance at 240 nm was measured by microplate reader (PerkinElmer Enspire). Enzyme activity was defined as the amount of enzyme required to oxidize 1μmol Mn^2+^ per minute. The enzymatic activity of MnP was calculated according to the following formula. The meanings of each item in the formula were the same as those in the laccase formula. Molar extinction coefficient of Mn^3+^-lactate is 7800M^-1^∙cm^-1^ at 240nm [14, 15].


$$U/L=\frac{\Delta OD\times V_{1}}{\varepsilon_{240}\times V_{2}\times \Delta t}\times 10^{6}$$

#### Determination of β-glucosidase

A volume of 1.5 mL of 3% salicin solution in 50mmol∙L^-1^citric acid buffer (pH 5.0) as reaction substrate and 1 mL crude enzyme solution were added to 25 mL plugged test tube, bathed in water at 50°C for 30 minutes. The above samples were added with 3 mL DNS, boiled for 10 minutes, cooled to room temperature in flowing water, added distilled water to the scale line, shaken well, and then added 200 μL sample into 96-well plate to determine its absorbance at 540 nm by microplate reader (PerkinElmer Enspire). The crude enzyme solution boiled and inactivated for 10 minutes served as control [16]. The enzymatic activity of β-glucosidase was calculated according to the following formula. The meanings of each item in the formula were the same as those in the CMCase.


$$U/L=\frac{\frac{\Delta OD-n}{m}\times V_{0}\times \frac{V_{1}}{V_{2}}}{M_{\mathrm{glucose}}\times \Delta t\times V_{2}}$$

#### Preparation of standard curves

**(1) Standard curve preparation of xylose** The standard xylose of 0.1 g was dissolved in 50 mL citric acid-sodium citrate buffer solution. After stirring and dissolving, it was transferred into 100 mL volumetric bottle. The standard xylose solution of 1 mg·mL^-1^ was prepared by adding citric acid-sodium citrate buffer. Ten clean plugged test tubes were added with 0.1 mL, 0.2 mL, 0.3 mL, 0.4 mL, 0.5 mL, 0.6 mL, 0.7 mL and 0.8 mL standard solution respectively. Distilled water was added to make volume of 1 mL, followed by addition of 3 mL DNS reagent, and boiled for 10 minutes. Distilled water was added to make volume 25 mL after cooling to room temperature. 200 uL was added to 96-well enzyme plate to determine its absorbance at 540 nm by microplate reader (PerkinElmer Enspire). Standard curve was made with absorbance value as ordinate and xylose concentration as abscissa.

Standard curve of xylose linear regression was performed with absorbance (y) as ordinate and xylose content (x) as abscissa. The regression equation was obtained as follows: y = 0.2242x + 0.0135 (R^2^ = 0.9988, n = 8). The linear relationship between xylose and absorbance was good in the range of 0.1 mg·mL^-1^~0.8 mg·mL^-1^.

**(2) Standard curve preparation of glucose:** The standard curve of glucose was prepared in the same way as that of the above steps. Xylose was replaced by glucose.

Standard curve of glucose linear regression was performed with absorbance (y) as ordinate and glucose content (x) as abscissa. The regression equation was obtained as follows: y = 0.305x-0.0064 (R^2^ = 0.9991, n = 7). The linear relationship between glucose and absorbance was good in the range of 0.2 mg·mL^-1^~1.2 mg·mL^-1^

### Statistical analysis

SPSS Statistical 17.0 statistical software was used for data processing. Variance analysis was used for comparison between groups at P < 0.05. Bivariate correlation was determined for analysis.

## Results

### Changes of environmental factors

**Fig 4:**
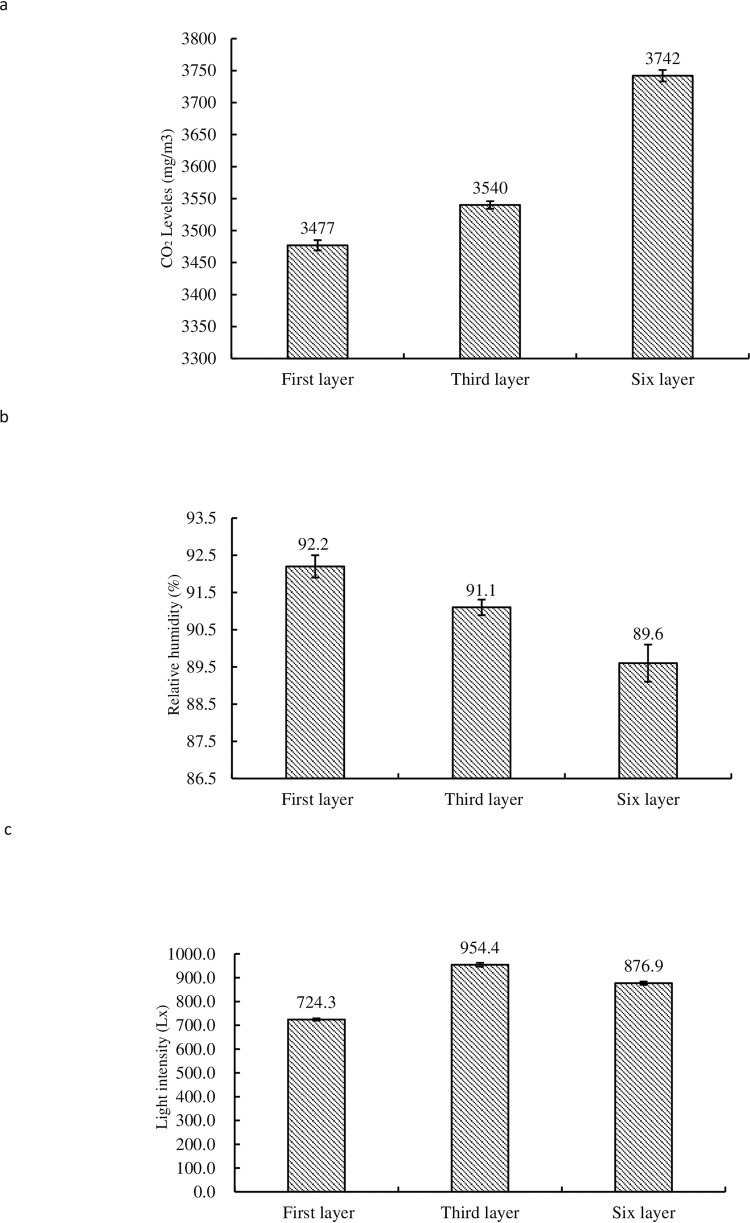
CO_2_ concentration (4a), humidity (4b) and light intensity (4c) in 1^st^, 3^rd^ and 6^th^ tier in mushroom house.

It can be seen from Fig 4A that from 1^st^ to 6^th^ tiers, the concentration of CO_2_ was on the rise, and 6^th^ tier was the strongest. The reason for the rising trend of CO_2_ concentration is may be due to the ventilator of the mushroom house on 1^st^ layer of the mushroom house. Humidity decreased gradually with the increase of tiers, and 6^th^ tier was slightly lower than 1^st^ and 3^rd^ tier (see Fig 4B). The intensity of light in 3^rd^ layer was the strongest, while that in 6^th^ layer was slightly higher than that in 1^st^ tier (see Fig 4C). Statistical analysis showed that there were significant differences in CO_2_ concentration and light intensity between tiers 1^st^, 3^rd^ and 6^th^ (P < 0.01), and there were significant differences in humidity between tiers1^st^, 3^rd^ and 6^th^ (P < 0.05).

### Xylanase activity assay

**Fig 5:**
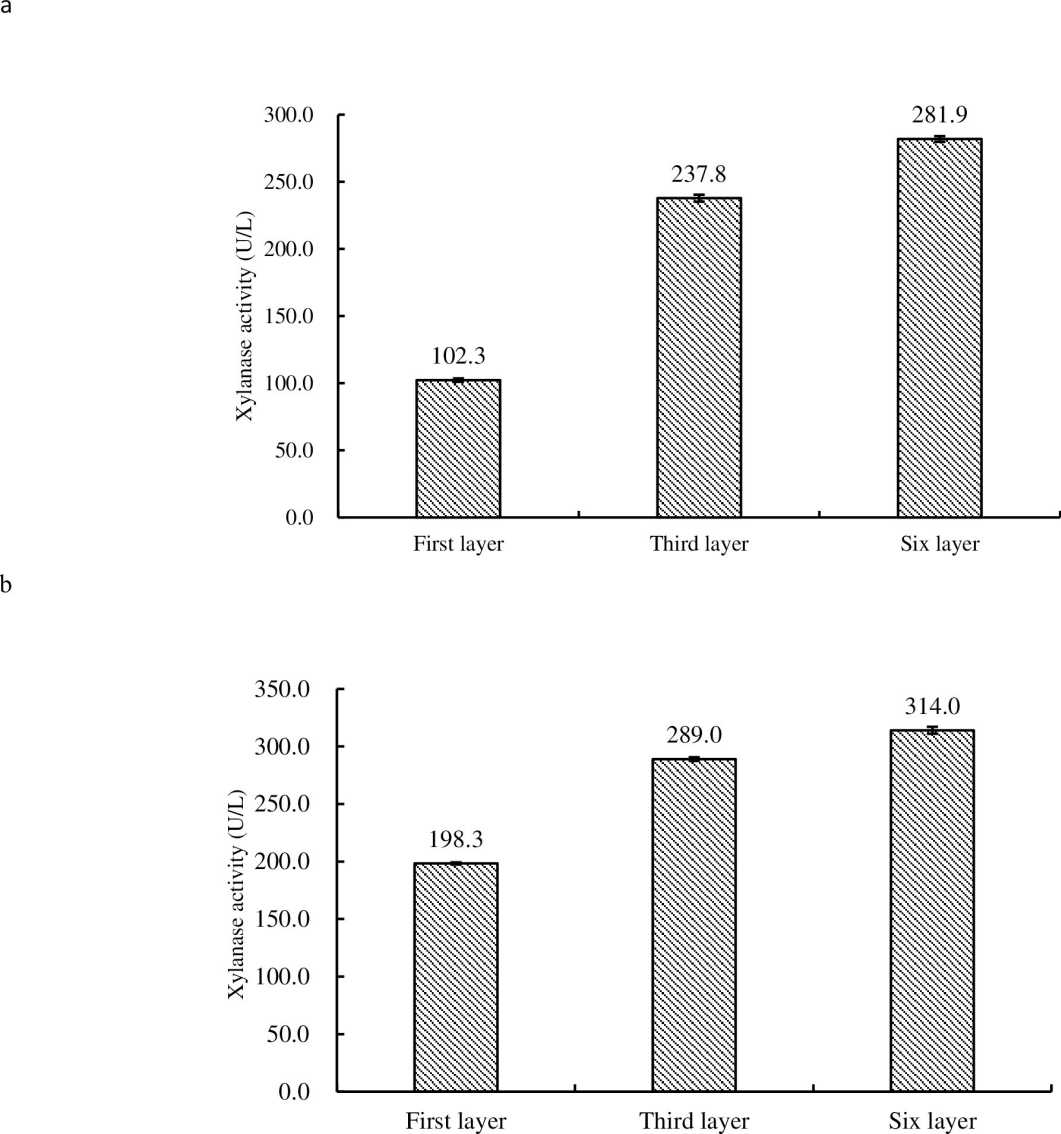
Comparison of xylanase activity in budding fruiting bodies (5a) and mature fruiting bodies (5b) of 1^st^, 3^rd^ and 6^th^ tier of *H*. *marmoreus*.

The data showed that the xylanase activity in fruiting bodies of *H*. *marmoreus* increased with the increase of sampling tiers. The highest xylanase activity was found in fruiting bodies of tier 6 i.e. in mature fruiting bodies 314.0 U/L and in buds 281.9 U/L (see Fig 5). Statistical analysis showed that there were significant differences in xylanase activity between 1^st^, 3^rd^ and 6^th^ tier in budding and mature fruiting bodies (P<0.01).

### Carboxymethyl cellulase assay

**Fig 6:**
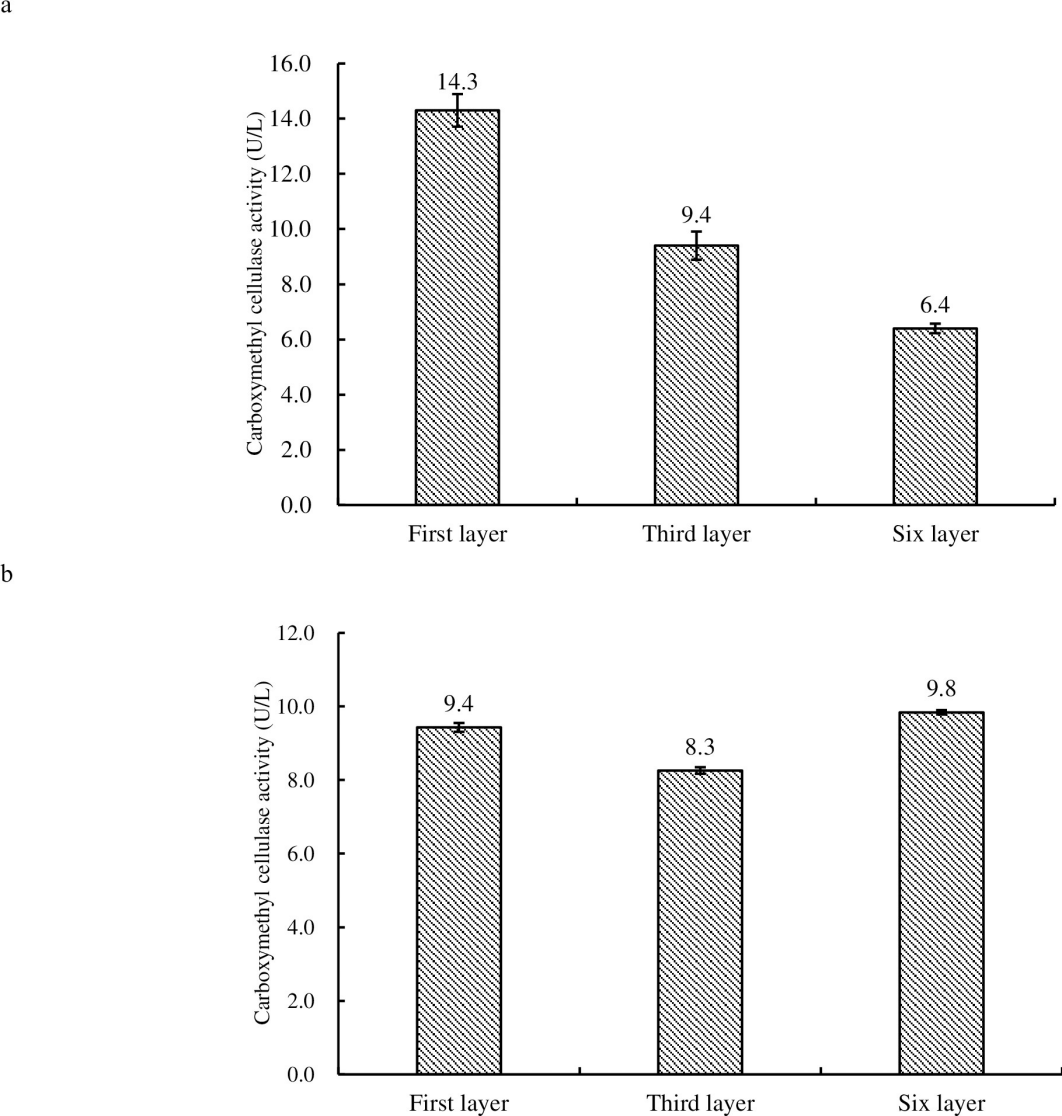
Comparison of CMCase activity in budding fruiting bodies (6a) and mature fruiting bodies (6b) of 1^st^, 3^rd^ and 6^th^ tier of *H*. *marmoreus*.

The enzyme activity in budding fruiting bodies of 1^st^ tier was the highest (14.3 U/L), while that of 6^th^ tier was the lowest (6.4 U/L) (see Fig 6A), yet, that in the mature fruiting bodies of 6^th^ tier (9.8U/L) was highest (see Fig 6B). Significant differences in CMCase activity between 1^st^, 3^rd^ and 6^th^ tier in the budded and mature fruiting bodies could be observed (P<0.01).

### Laccase assay

**Fig 7:**
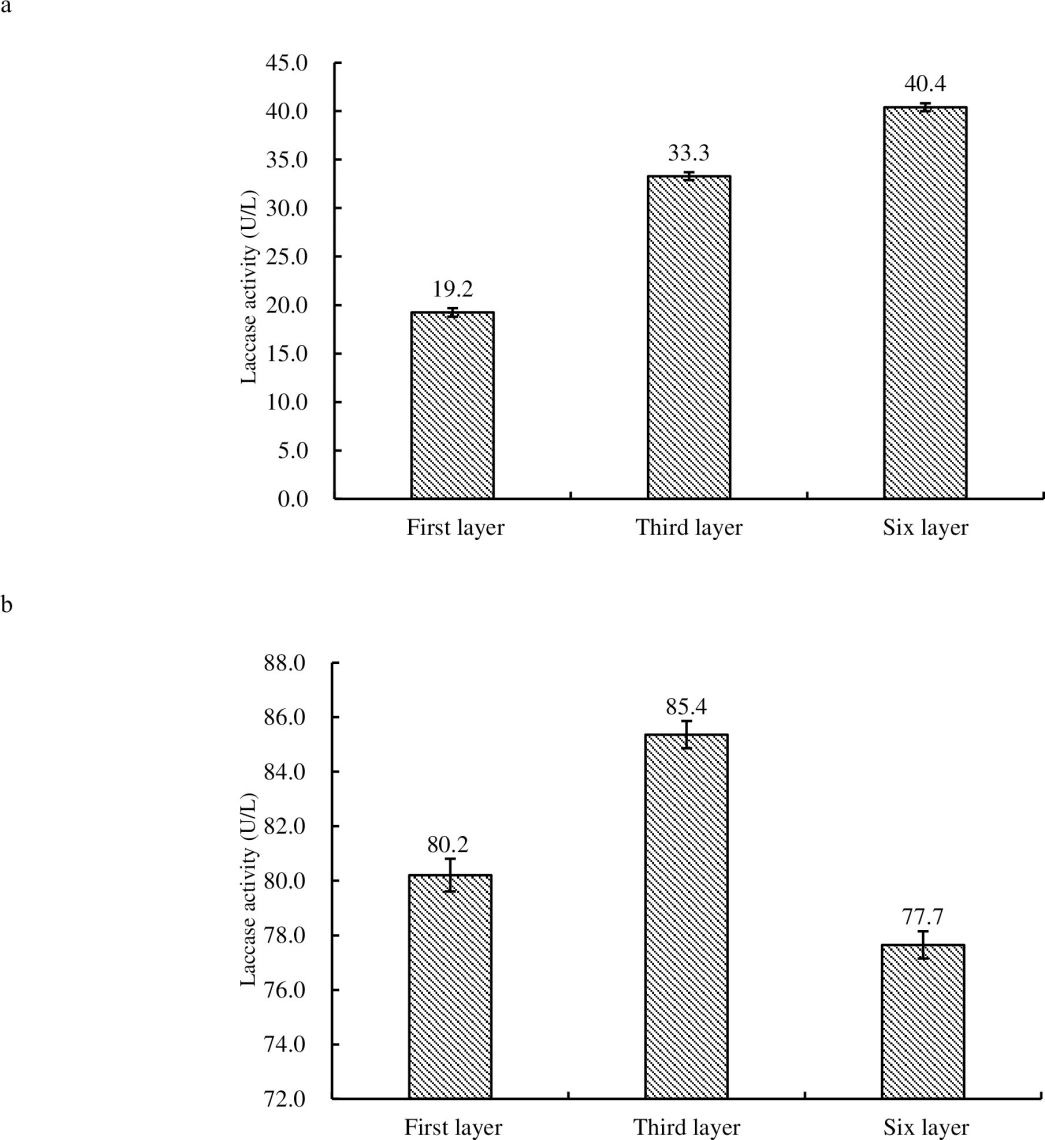
Comparison of laccase activity in budding fruiting bodies (7a) and mature fruiting bodies (7b) of 1^st^, 3^rd^ and 6^th^ tier of *H*. *marmoreus*.

Laccase activity in budding fruiting bodies of *H*. *marmoreus* of 6^th^ tier was the highest (40.4 U/L) (see Fig 7A), while that of 1^st^ tier was the lowest (19.2 U/L) yet, that in the mature fruiting bodies of 6^th^ tier (77.7U/L) was lowest (see Fig 7B). Laccase activity showed significant variation in budding and mature fruiting bodies of 1^st^, 3^rd^ and 6^th^ tier (P<0.01).

### Manganese peroxidase assay

**Fig 8:**
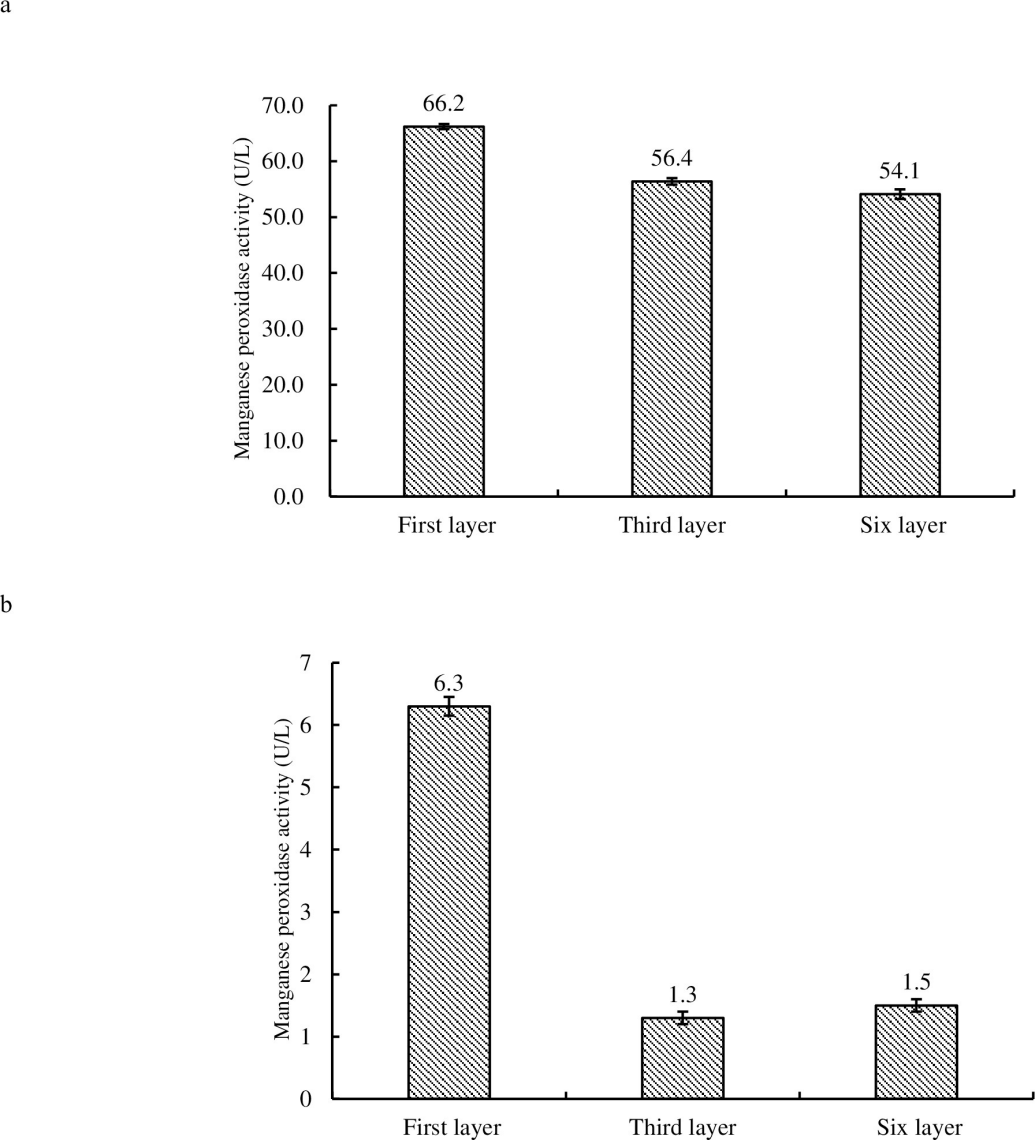
Comparison of MnP activity in budding fruiting bodies (8a) and mature fruiting bodies (8b) of 1^st^, 3^rd^ and 6^th^ tier layer of *H*. *marmoreus*.

The maximum MnP activity (66.2 U/L) was observed in budding fruiting bodies of 1^st^ tier (see Fig 8A), while that of 6^th^ tier was the lowest (54.1 U/L). In matured fruiting bodies, the MnP activity was observed to be the highest in 1^st^ tier (6.3U/L) (see Fig 8B). It was also observed that the MnP activity in the mature fruiting body of 1^st^ tier significantly differed from that in 3^rd^ tier and 6^th^ tier (P < 0.01) while no significant difference was observed between 3^rd^ and 6^th^ tier. Moreover, the MnP activity of the mature fruiting bodies from *H*. *marmoreus* was very low with respect to budding fruiting bodies.

### β-glucosidase assay

**Fig 9:**
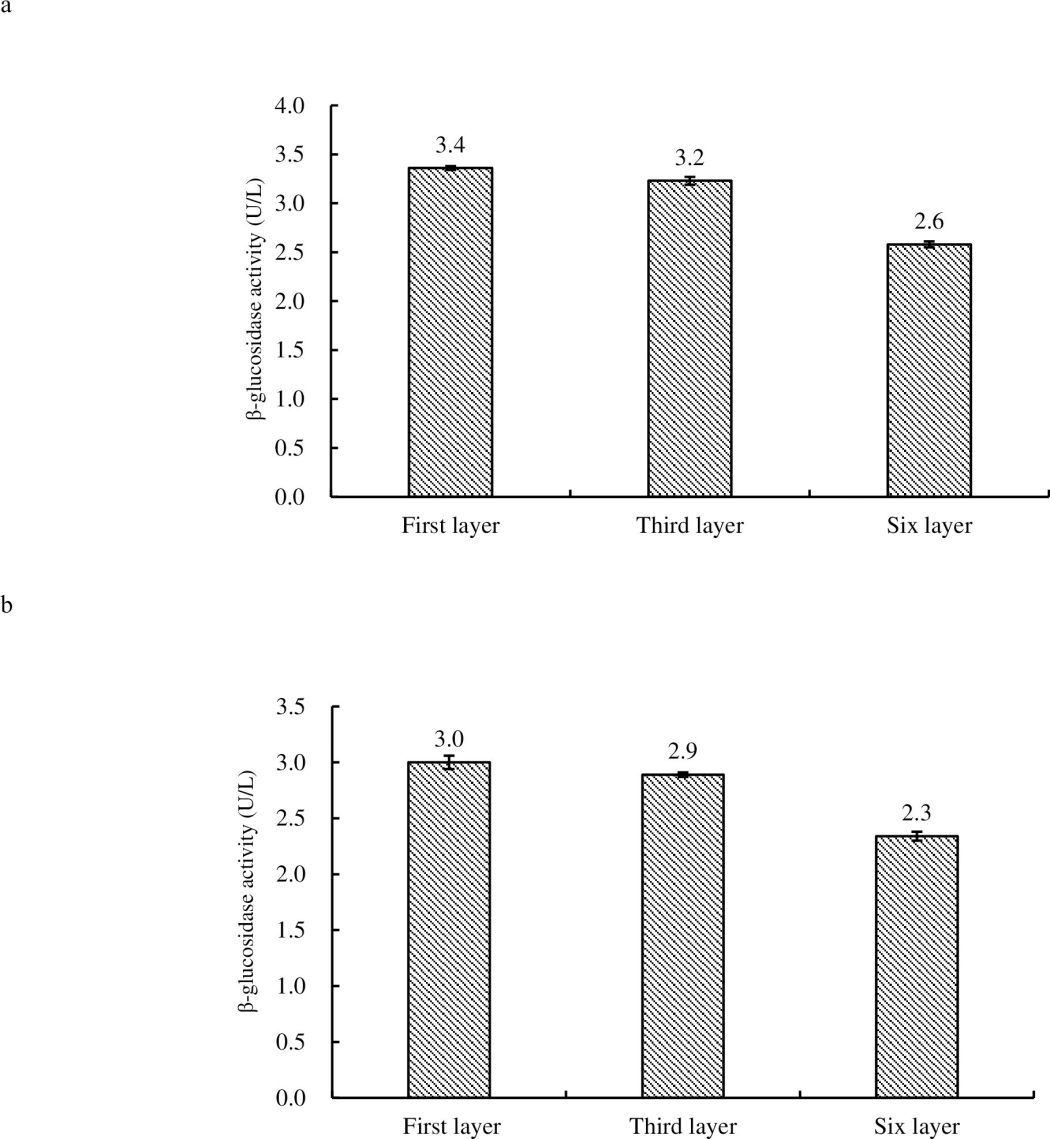
Comparison of β-glucosidase activity in budding fruiting bodies (9a) and mature fruiting bodies (9b) of 1^st^, 3^rd^ tier and 6^th^ tier of *H*. *marmoreus*.

β-glucosidase activity of budding fruiting bodies and mature fruiting bodies in 1^st^ layer was the highest (3.4 U/L and 3.0 U/L respectively), and in 6^th^ was the lowest (2.3 U/L) (see Fig 9A and 9B). The enzyme activity differed significantly among 1^st^, 3^rd^ and 6^th^ tiers in the budded fruiting bodies (P<0.05; P<0.01) whereas significant differences were observed in mature fruiting bodies of 1^st^ and 6^th^ tier, and between 3^rd^ tier and 6^th^ tier (P<0.01). There was no significant difference in the activity of β-glucosidase between mature fruiting bodies of 1^st^ and 3^rd^ tier.

### Correlation analysis of CO_2_ and five enzyme activities

Correlation analysis showed that there was a significant positive correlation between xylanase activity and CO_2_ concentration in the budding and mature fruiting bodies (P<0.01). CMCase activity and MnP activity in budding fruiting bodies were significantly negatively correlated with CO_2_ concentration (P<0.01). Laccase activity was markedly positively correlated with CO_2_ concentration in the budding fruiting bodies (P<0.01). The activity of β-glucosidase in the budding and in the mature fruiting bodies was significantly negatively correlated with CO_2_ concentration (P<0.01). CMCase activity of mature fruiting bodies was positively correlated with CO_2_ concentration. Laccase and MnP activities of mature fruiting bodies were negatively correlated with CO_2_ concentration (see Table 2).

**Table 2:** The result on correlation analysis of CO_2_ and five enzymes.

Control variable		XylB	XylM	CMCB	CMCM	LacB	LacM	MPB	MPM	β-gluB	β-gluM
CO_2_	correlation	0.837**	0.819**	-0.940**	0.520	0.876**	-0.585	-0.827**	-0.658	-0.993**	-0.984**
	significance	0.005	0.007	0.000	0.151	0.002	0.098	0.006	0.054	0.000	0.000

a. Xylan of budding fruiting body (XylB); Xylan of mature fruiting body (XylM); CMCase of budding fruiting body (CMCB); CMCase of mature fruiting body (CMCM); Laccase of budding fruiting body (LacB); Laccase of mature fruiting body (LacM); MnP of budding fruiting body (MPB); MnP of mature fruiting body (MPM); β-glucosidase of budding fruiting body (β-gluB); β-glucosidase of mature fruiting body (β-gluM)

**At the level of 0.01 (bilateral), there was a significant correlation

*At the level of 0.05 (bilateral), there was a significant correlation.。

### Correlation analysis of humidity and five enzyme activities

Correlation analysis showed that there was a significant negative correlation between xylanase activity and humidity in the budding and mature fruiting bodies (P<0.01). MnP activity and β-glucosidase activity of budding and mature fruiting bodies were dramatically positively correlated with humidity (P<0.01, P<0.05). CMCase activity in the budding fruiting bodies was significantly positively correlated with humidity (P<0.01) while negatively correlated in mature fruiting bodies. Laccase activity was significantly negatively correlated with humidity in the budding fruiting bodies (P<0.01), while positively correlated in mature fruiting bodies (see Table 3).

**Table 3:** The result on correlation analysis of humidity and five enzymes.

Control variable		XylB	XylM	CMCB	CMCM	LacB	LacM	MPB	MPM	β-gluB	β-gluM
Relative humidity	correlation	-0.889**	-0.879**	0.949**	-0.356	-0.914**	0.424	0.880**	0.751*	0.946**	0.876**
	significance	0.001	0.002	0.000	0.347	0.001	0.255	0.002	0.020	0.000	0.002

a. Xylan of budding fruiting body (XylB); Xylan of mature fruiting body (XylM); CMCase of budding fruiting body (CMCB); CMCase of mature fruiting body (CMCM); Laccase of budding fruiting body (LacB); Laccase of mature fruiting body (LacM); MnP of budding fruiting body (MPB); MnP of mature fruiting body (MPM); β-glucosidase of budding fruiting body (β-gluB); β-glucosidase of mature fruiting body (β-gluM)

**At the level of 0.01 (bilateral), there was a significant correlation

*At the level of 0.05 (bilateral), there was a significant correlation.。

### Correlation analysis of light intensity and five enzymes activities

Significant positive correlation was observed between xylanase activity and light intensity in the budding and mature fruiting bodies (P<0.01) (see Table 4). CMCase activity in the budding fruiting bodies was markedly negatively correlated with light intensity (P<0.05), while was not markedly negatively correlated in mature fruiting bodies. Laccase activity in the budding fruiting bodies was remarkably positively correlated with light intensity (P<0.05), while positively correlated in mature fruiting bodies. The MnP activity in the budding and mature fruiting bodies was significantly negatively correlated with light intensity (P<0.01). β-glucosidase activity in the budding and mature fruiting bodies was negatively correlated with light intensity (P<0.05). (see Table 4).

**Table 4:** The result on correlation analysis of light intensity and five enzymes.

Control variable		XylB	XylM	CMCB	CMCM	LacB	LacM	MPB	MPM	β-gluB	β-gluM
Light intensity	correlation	0.829**	0.845**	-0.682*	-0.578	0.785*	0.512	-0.839**	-0.946**	-0.327	-0.210
	significance	0.006	0.004	0.043	0.103	0.012	0.159	0.005	0.000	0.391	0.587

a. Xylan of budding fruiting body (XylB); Xylan of mature fruiting body (XylM); CMCase of budding fruiting body (CMCB); CMCase of mature fruiting body (CMCM); Laccase of budding fruiting body (LacB); Laccase of mature fruiting body (LacM); MnP of budding fruiting body (MPB); MnP of mature fruiting body (MPM); β-glucosidase of budding fruiting body (β-gluB); β-glucosidase of mature fruiting body (β-gluM)

**At the level of 0.01 (bilateral), there was a significant correlation

*At the level of 0.05 (bilateral), there was a significant correlation.

## Discussion

The main environmental factors of mushroom growth and development include temperature, humidity, luminosity and the air composition of the surrounding matrix, such as the concentration of oxygen and carbon dioxide [4, 17]. Mushroom absorbs its nutrients from the substrate (grasses, wood and agricultural residues) through its mycelium, obtaining substances necessary for its development, such as carbon, nitrogen, vitamins and minerals [18]. The utilization of different types of substrate by fungus (mushroom) will depend on its capacity to secrete enzymes such as oxidative (ligninase, laccase, MnP) and hydrolytic (cellulase, xylanase and tannase) enzymes, which are involved in utilizing lignocellulosic substrates [4, 19]. In this research, it was found that the enzymatic activity of *H*. *marmoreus* in the growth process was related to the light intensity, humidity and CO_2_ concentration in the growth environment. It is important to keep the CO_2_ concentration in a certain range during the darkened spawn-running and fruiting phases. If the growth environment contains high concentration of CO_2_, the mushroom with thick and short stipe pileus will be produced in the growth process [6, 18]. The appearance of mushroom is closely related to its growth process, and the changes of enzyme activities related to the growth and development of mushroom are the deep-seated reasons.

Xylanase plays a role in decomposing hemicellulose (xylan) in cultivation material during the growth of mushroom [20, 21], helping fungal mycelium to utilize nutrients in cultivation material and make fruiting body grow. The results showed that CO_2_ and light intensity have a positive effect on xylanase activity in a certain concentration range, while relative humidity has a negative effect. It could be inferred from the present study that the xylanase activity of fruiting bodies at ripening stage was significantly higher than that at budding stage, suggesting that xylanase accumulated in fruiting bodies during the development of fruiting bodies. The result is consistent with the literature report from *Flammulina velutipes* [21]. This indicated that xylanase played an important role in the growth and development of mushrooms in general and *H*. *marmoreus* in particular.

CMCase converts cellulose to cellobiose and glucose. Cellobiose and other cello- oligosaccharides could be hydrolyzed by extracellular or intracellular β-glucosidase after cellobiose is transported into the cell by cellodextrin transporters [22, 23]. Results obtained in this study indicated that light intensity have a negative effect on CMCase and β-glucosidase. At budding stage, carbon dioxide had a negative effect on CMCase activity while relative humidity had a positive effect. Carbon dioxide had a negative effect on β-glucosidase while relative humidity had a positive effect. Therefore, relatively low CO_2_ concentration, light intensity and relatively high relative humidity may be conducive for the accumulation of CMCase in the budding stage of *H*. *marmoreus*. In addition, compared with xylanase, CMCase, laccase and MnP, the activity of β-glucosidase in the fruiting body of *H*. *marmoreus* was lower during the growth and development. It could also be inferred that the activity of β-glucosidase in *H*. *marmoreus* was lower than others mushroom [21].

In fungi, laccase is known as a ligninolytic enzyme and appears to be involved in fruiting body formation [23]. The lignin in the culture medium was hydrolyzed by laccase and MnP. In this research, the results indicated that laccase was gradually accumulated or increased in the fruiting body of *H*. *marmoreus* with the growth phases. At budding stage, Carbon dioxide and light intensity had a positive effect while relative humidity had a negative effect on laccase. Comparing the activity of MnP in mature fruiting bodies with that in budding fruiting bodies of *H*. *marmoreus*, the activity of MnP in mature fruiting bodies was too low. The reason may be that the fruiting bodies of *H*. *marmoreus* do not need MnP to provide nutrition at this physiological stage of fruiting. During the growth and development of *H*. *marmoreus*, carbon dioxide and light intensity had a negative effect on MnP while relative humidity had a positive effect. In budding stage, the CO_2_, relative humidity and light intensity had an inconsistent correlation. This reason needs to be further studied.

In this study, it can be seen that the enzyme activity differed depending on different environmental conditions tested in this study. The reason for these changes may be attributed to the influence of environmental factors on cell respiration during the growth and development, which affected the expression of these enzymes. In particular, because the ventilation fan in the mushroom house is only 35cm above the ground, the air flow, CO_2_ and O_2_ concentrations are different at different heights and tiers of the mushroom rack resulting changes in enzyme activities. Therefore, it is very important to regulate and control the environmental factors CO_2_, relative humidity and light intensity in the cultivation of *H*. *marmoreus*, which will directly affect the growth and development and yield of *H*. *marmoreus*.
